# Recovery efficiency and functional characterization of T cells and NK cells from leukocyte filters

**DOI:** 10.3389/fmed.2025.1681146

**Published:** 2025-11-28

**Authors:** Liyan Sun, Ran Li, Siqi Cai, Songxing Wang, Jinfeng Zeng

**Affiliations:** 1 Institution of Transfusion Medicine, Shenzhen Blood Center, Shenzhen, Guangdong, China; 2 Department of Laboratory Medicine, Shenzhen Blood Center, Shenzhen, Guangdong, China; 3 Department of Blood Transfusion, The Eighth Affiliated Hospital of Sun Yat-sen University, Shenzhen, Guangdong, China; 4 Department of Laboratory Medicine, The Third People’s Hospital of Longgang District Shenzhen, Guangdong, China

**Keywords:** leukocyte filter, whole blood, NK cells, T cells, transcriptome sequencing

## Abstract

**Objective:**

This study aimed to investigate the recovery efficiency and functional characteristics of lymphocytes isolated from leukocyte filters, compared with those isolated directly from whole blood sample, and to assess their potential as an alternative immune cell source for therapeutic applications.

**Methods:**

From December 2023 to 2024, leukocyte filters and 5 mL whole blood samples were collected from healthy donors. Cell recovery was optimized by testing different flushing methods (forward/reverse), solutions (1 × PBS/NS), and volumes (20 – 80 mL). Peripheral blood mononuclear cells (PBMCs) were isolated via density gradient centrifugation, and lymphocyte subsets (CD3^+^CD56^+^ NK, CD3^+^CD4^+^ T, CD3^+^CD8^+^ T cells) were analyzed by flow cytometry. Transcriptome sequencing was conducted to evaluate functional differences.

**Results:**

Reverse flushing with 40 mL normal saline (NS) achieved cost-effective lymphocyte recovery, with cell counts correlating with flushing method and volume. The proportion of CD3^+^CD4^+^ T cells in 40 mL NS was higher than that in whole blood samples (*P* < 0.05), while CD3^+^CD8^+^ T cells was lower (*P* < 0.01). CD3^+^CD4^+^ T cells exhibited the highest recovery efficiency, and CD3^+^CD8^+^ T cells the lowest. Transcriptome analysis identified 634 differentially expressed genes, however, GO/KEGG enrichment analysis revealed no significant pathways related to NK or T cell functions (*P*-adjust > 0.05).

**Conclusion:**

Reverse flushing of leukocyte filters with 40 mL NS enables cost-effective recovery of lymphocytes, particularly CD3^+^CD4^+^ T cells. The recovered NK and T cells exhibit functional equivalence to those from those from whole blood samples, offering a low-cost and sustainable cell source for allogeneic cell therapy.

## Background

Human peripheral blood cells are a critical source of primary cells for basic and preclinical research. Among these, immune cells play a pivotal role in investigating immune mechanisms and developing cell therapies, particularly T cells and NK cells (1–3). Currently, these cells are primarily obtained by recruiting healthy individuals, a process that is resource-intensive and often fails to satisfy the growing demand for research and clinical applications (4, 5), resulting in shortages of healthy immune cell sources.

In China, most blood banks use leukocyte filters to remove white blood cells (WBC) from whole blood before transfusion, minimizing the risk of immune rejection in recipients (6, 7). For instance, in 2024, approximately 99,127 leukocyte filters were used for 183,219.25 units of whole blood at the Shenzhen Blood Center; these filters were typically discarded as medical waste after use (8). Recovering immune cells from leukocyte filters as a potential alternative source has attracted the researchers’ attention.

While numerous studies have investigated cell recovery from leukocyte filters, their findings are not consistent. The primary reason for this discrepancy lies in the different types of filters used. Specifically, the effectiveness of reverse flushing or a sequential forward-then-reverse flushing protocol has been shown to vary across different filter models (9–11). In addition, numerous studies have identified the types and proportions of NK or T cells recovered from leukocyte filters (12, 13), and some have even demonstrated the therapeutic potential of *in vitro*-expanded NK or T cells derived from them (14–16), a comparative analysis of the quantity and function of these cells against their counterparts isolated directly from whole blood is still lacking.

Sourcing cells from used leukocyte filters is an emerging strategy globally to reduce biological waste and provide a cost-effective cell source, though standardization is needed. Based on this, this study investigates cost-effective conditions for recovering immune cells from leukocyte filters and compares their functionality with those from whole blood samples, aiming to provide NK and T cells for cell therapy. Previous studies suggest that 40–50 mL of whole blood provides an adequate number of cells for therapeutic use and meets the input requirements of various NK and T cell culture systems (17, 18). Therefore, we used 40 mL as the benchmark volume for comparing cell yields from filters and whole blood.

## Materials and methods

### Sample collection

From December 2023 to December 2024, leukocyte filters (NGF/RL-XZ Q400, Sichuan Nangel Biotechnology Co., Ltd., China) and 5 mL EDTA-anticoagulated whole blood were collected from healthy blood donors at the Shenzhen Blood Center. Exclusion criteria included first-time donation, positive results for transfusion-transmitted diseases, donation volume < 400 mL, and refusal to sign informed consent. The leukocyte filters used in this study process 400 mL of whole blood. All leukocyte filters and whole blood samples were processed within 24 h of collection. During temporary storage, samples were kept at room temperature (20 °C–25 °C) in a sterile environment to maintain cell viability and functional integrity. The study was approved by the Ethics Committee of the Shenzhen Blood Center (SZBC-2023-R008).

### Leukocyte filter flushing

**FIGURE 1:**
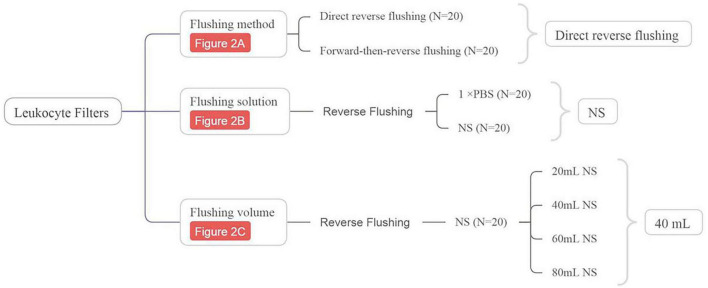
Schematic of the leukocyte filter flushing strategy.

A schematic diagram illustrating the flushing strategies and their association with specific figures has been included as Figure 1, highlighting the optimal strategy.

#### Flushing methods

Twenty of the leukocyte filters were flushed in the direct reverse direction (hereafter referred to as “direct reverse flushing”), and 20 leukocyte filters were flushed in the forward direction followed by reverse flushing (hereafter referred to as “forward-then-reverse flushing”), all with 1 × PBS (1× Phosphate-Buffered Saline, Beijing Solarbio Science & Technology Co., Ltd., China). Cell counts were determined using an automated cell counter (BC-30S, Shenzhen Mindray Bio-Medical Electronics Co., Ltd., China), and lymphocyte counts in the two filter groups were compared to the calculated lymphocyte count in 40 mL of whole blood. The lymphocyte count for 40 mL whole blood was derived by proportionally scaling the count from the 5 mL donor sample (i.e., Lymphocyte count in 5 mL WB × 8).

#### Flushing solutions

Twenty of the leukocyte filters were flushed with NS (Sterile 0.9% Sodium Chloride Solution, Guangdong Kelun Pharmaceutical Co., LTD., China) in the direct reverse direction, and 20 were flushed with 1 × PBS in the direct reverse direction. cell counts were determined using an automated cell counter, and lymphocyte counts in 1 × PBS and NS were compared to the calculated lymphocyte count in 40 mL of whole blood (derived as above).

#### Flushing volumes

Twenty leukocyte filters were flushed in the direct reverse with NS, with 20 mL fractions collected. Lymphocyte counts in 20, 40, 60, and 80 mL NS were compared to the calculated lymphocyte counts in equivalent volumes (20, 40, 60, 80 mL) of whole blood. The lymphocyte counts for these different whole blood volumes were calculated by proportionally scaling the cell count obtained from the standard 5 mL whole blood sample (e.g., for 20 mL WB: Lymphocyte count in 5 mL WB × 4; for 40 mL WB: Lymphocyte count in 5 mL WB × 8, etc.).

### PBMC isolation

Peripheral blood mononuclear cells were isolated from leukocyte filters (*N* = 85) and 5 mL EDTA-anticoagulated whole blood. Briefly, samples were diluted 1:1 with 1 × PBS, carefully layered over 3.5 mL Lymphoprep density gradient medium (STEMCELL Technologies, Canada) in SepMate PBMC Isolation Tubes (STEMCELL Technologies. Canada), and centrifuged at 1200 × *g* for 10 min at room temperature (20 °C–25 °C). The upper plasma layer was aspirated, and the PBMC-rich interface was collected, washed once with 1 × PBS, and centrifuged at 300 × *g* for 8 min. The resulting cell pellet was resuspended in 1 × PBS, and viable cell counts were determined using an automated cell counter (BC-30S, Shenzhen Mindray Bio-Medical Electronics Co., Ltd., China).

### Flow cytometry

For immunophenotyping analysis, PBMCs (1 × 10^6^ cells per sample) were stained with the following antibody cocktail: 5 μL each of FITC-anti-CD3, PE/Cy7-anti-CD56, APC-anti-CD4, and PE-anti-CD8 antibodies (BioLegend, Inc., USA) in the experimental group. Appropriate isotype controls (BioLegend, Inc., USA) and an unstained negative control (blank) were included for gating and compensation purposes. After incubation in the dark at room temperature for 30 min, cells were washed three times with 1 × PBS, resuspended in staining buffer, and subsequently analyzed by Attune NxT Flow Cytometer (Thermo Fisher Scientific, USA).

### RNA extraction and transcriptome sequencing

Peripheral blood mononuclear cells used for RNA extraction were isolated from leukocyte filters via direct reverse flushing with 40 mL NS. At least 1 × 10^6^ PBMCs were used for RNA extraction. PBMCs from 12 donors (isolated from both leukocyte filters and EDTA-anticoagulated whole blood) were lysed in 1 mL QIAzol Lysis reagent (Qiagen GmbH, Germany), followed by the addition of 200 μL chloroform. After phase separation by centifugation, the aqueous phase was collected and mixed with 900 μL of 100% ethanol. The mixture was then processed using the miRNeasy Serum/Plasma Kit (Qiagen GmbH, Germany) according to the manufacturer’s instructions, including washes with Buffer RWT and twice with Buffer RPE. Finally, total RNA was eluted in 35 μL of RNase-free water. RNA concentration was quantified using a NanoDrop spectrophotometry (A260/A280 ratio > 1.8) and agarose gel electrophoresis.

For transcriptome sequencing, Poly(A) + mRNA was enriched using oligo (dT) magnetic beads, chemically fragmented, and reverse-transcribed into double-stranded cDNA. The resulting libraries were purified (AMPure XP beads), size-selected (200–500 bp), and PCR-amplified. Paired-end sequencing (150 bp) was performed on an Illumina NovaSeq 6000 platform.

### Statistical analysis

Statistical analysis was performed using Graphpad Prism 10.4.0 software. First, the Shapiro-Wilk test is used to assess whether the data follow a normal distribution. If the data are normally distributed and meet the assumption of homogeneity of variance (*F*-test), parametric tests can be used for group comparisons: an independent samples *t*-test for two-group comparisons, or one ANOVA for multiple groups. If ANOVA reveals significant differences, Welch ANOVA test should be conducted.

If the data do not follow a normal distribution, non-parametric tests should be employed: the Mann-Whitney U test for two independent groups, or the Kruskal-Wallis H test for multiple independent groups. If the Kruskal-Wallis test indicates significant differences, Dunn’s test can be performed.

FlowJo v10.9 software was used to analyze the results of flow cytometry. For transcriptome data, differential gene expression analysis was performed with DESeq2, applying thresholds of |log_2_ fold change| > 1 and false discovery rate (FDR) < 0.05. Functional enrichment analysis of differentially expressed genes was conducted via Gene Ontology (GO) and Kyoto Encyclopedia of Genes and Genomes (KEGG) pathway analyses using clusterProfiler.

## Results

### Impact of flushing method, volume, and solution

**FIGURE 2:**
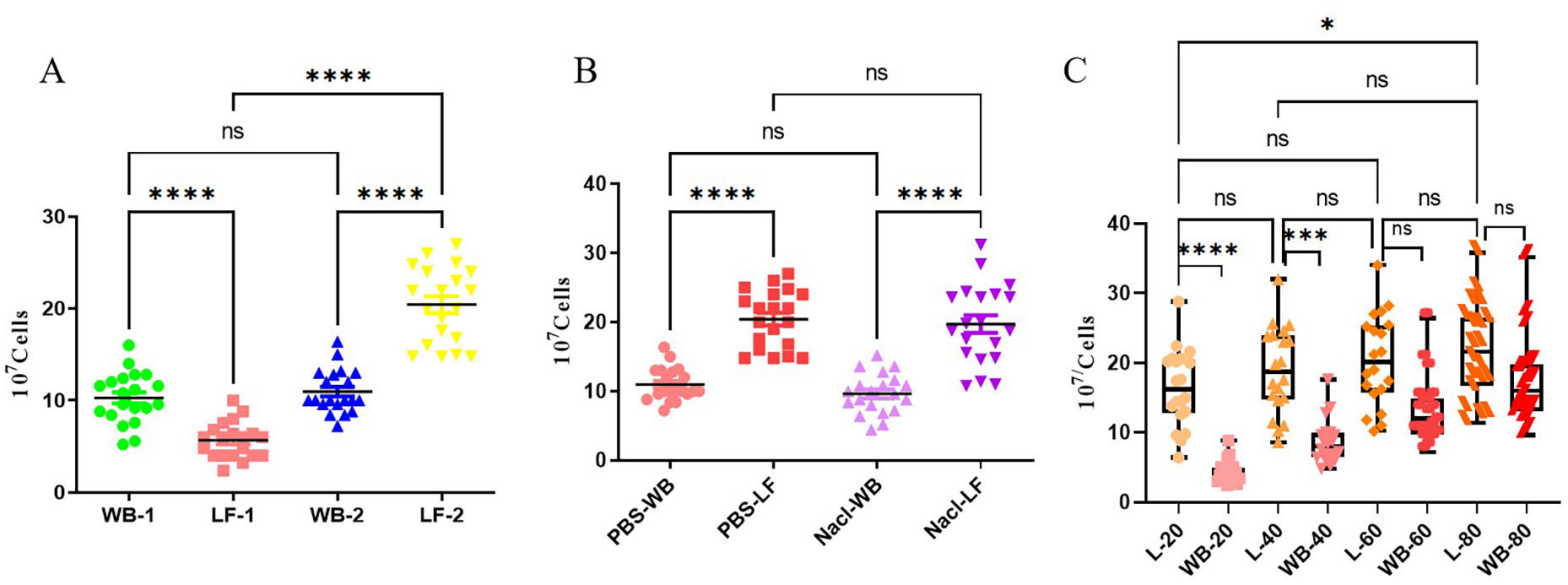
Effects of different flushing methods, flushing solutions and flushing volumes on the number of lymphocytes recovered from leukocyte filters. **(A)** Comparison between the direct reverse flushing (LF-2) and the forward-then-reverse flushing (LF-1) (*N* = 20 per group); **(B)** comparison between reverse flushing with 1 × PBS (PBS-LF) and NS (NS-LF) (*N* = 20 per group); **(C)** comparison of reverse flushing with different volumes of NS (*N* = 20); LF, leukocyte filter; WB, whole blood sample. WB-1 and WB-2 refer to the whole blood samples corresponding to the forward-then-reverse flushing and direct reverse flushing groups, respectively. ns, not significant (*P* > 0.05); *, ***, **** indicate *P* < 0.05, 0.001, and 0.0001, respectively.

When comparing two flushing methods (direct reverse flushing vs. forward flushing followed by reverse flushing), the number of lymphocytes in the 40 mL eluates obtained by forward-then-reverse flushing was significantly lower than that in the 40 mL whole blood (WB-1 vs. LF-1, *P* < 0.0001; Figure 2A), while that by direct reverse flushing was significantly higher than that in the 40 mL whole blood (WB-2 vs. LF-2, *P* < 0.0001; Figure 2A). Furthermore, a direct comparison between the two flushing methods revealed that direct reverse flushing yielded significantly more lymphocytes than forward-then-reverse flushing (LF-2 vs. LF-1, *P* < 0.0001; Figure 2A). There was no significant statistical difference in lymphocyte counts between two groups’ whole blood samples (WB-1 vs. WB-2, *P* > 0.05; Figure 2A).

In solution comparison tests, we observed no significant difference in lymphocyte recovery between 40 mL 1 × PBS and 40 mL NS when using reverse flushing (PBS-LF vs. NS-LF, *P* = 0.95 > 0.05; Figure 2B). Similarly, lymphocytes from whole blood samples counts did not differ significantly between the two solution groups (PBS-WB vs. NS-WB, *P* = 0.72 > 0.05; Figure 2B).

For flushing volumes, no significant difference was found between 20 and 40 mL eluates (L-20 vs. L-40, *P* = 0.57 > 0.05; Figure 2C), but 20 mL eluates had fewer lymphocytes than 80 mL eluates (L-20 vs. L-80, *P* < 0.05; Figure 2C). No significant difference was observed between 40, 60, and 80 mL eluates (L-40 vs. L-60 vs. L-80, *P* = 0.36 > 0.05; Figure 2C). Although recovery increased with volume up to 80 mL without a clear plateau, 40 mL was chosen for subsequent experiments as it provided a cost-effective yield not significantly different from 60 to 80 mL, while using less reagent.

### Lymphocyte subset proportions and recovery efficiency

**FIGURE 3:**
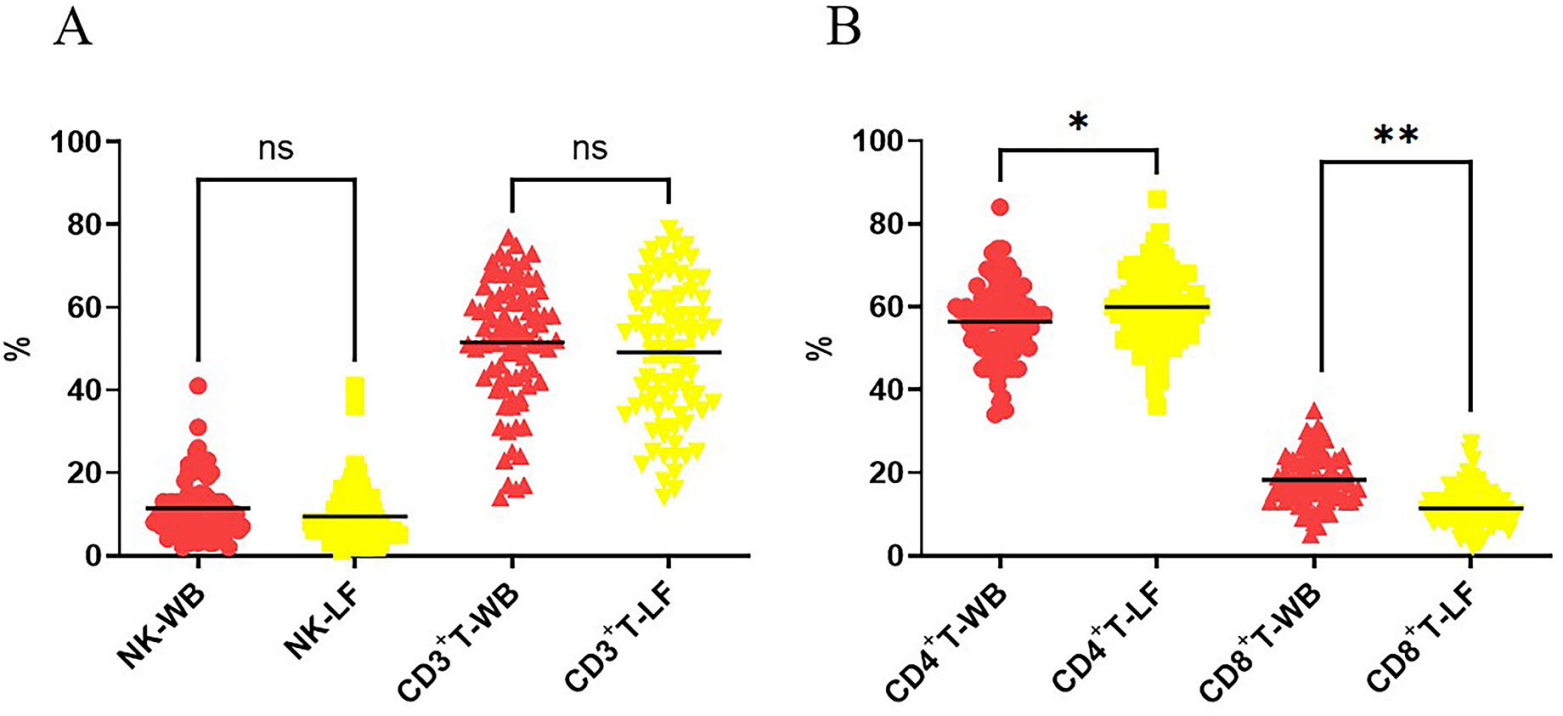
Comparison of the proportions of lymphocyte subsets between leukocyte filter eluates and whole blood samples. **(A)** Comparison of the proportions of NK cells and T cells between leukocyte filter eluates and whole blood samples. **(B)** Comparion of the proportions of CD3^+^CD4^+^ T cellls and CD3^+^CD8^+^ T cells between leukocyte filter eluates and whole blood samples. NK-LF refers to CD3CD56^+^ NK cells derived from filters, NK-WB refers to CD3CD56^+^ NK cells derived from whole blood; CD3^+^T-LF refers to CD3^+^ T cells from leukocyte filters, CD3^+^T-WB refers to CD3^+^ T cells from whole blood samples; CD4^+^T-LF refers to CD3^+^CD4^+^ T cells from leukocyte filters, CD4^+^T-WB refers to CD3^+^CD4^+^ T cells from whole blood samples; CD8^+^T-LF refers to CD3^+^CD8^+^ T cells from leukocyte filters, CD8^+^T-WB refers to CD3^+^CD8^+^ T cells from whole blood samples; The percentages of CD3^+^CD4^+^ T and CD3^+^CD8^+^ T cells are based on the total CD3^+^ T cell population. ns, not significant (*P* > 0.05); *N* = 85, * indicates *P* < 0.05, ** indicates *P* < 0.01.

A representative flow cytometry plot illustrating the gating strategy used to identify lymphocyte subsets is provided in Supplementary Figure 1. The results of flow cytometry showed (Figure 3, *N* = 85) that there was no significant difference in the proportions of NK cells and CD3^+^ T cells between 40 mL eluates and whole blood (NK-WB vs. NK-LF, *P* > 0.99; T-WB vs. T-LF, *P* > 0.99; Figure 3). However, we observed significant subset-specific variations among T cell populations. The proportion of CD3^+^CD4^+^ T cells in eluates was significantly higher than that in whole blood sample (CD4^+^T-LF vs. CD4^+^T-WB, *P* < 0.05; Figure 3), while the proportion of CD3^+^CD8^+^ T cells was significantly lower than that in whole blood sample (*P* < 0.01). (CD8^+^T-LF vs. CD8^+^T-WB, *P* < 0.01; Figure 3). These findings suggest selective recovery of specific lymphocyte subsets during the elution process.

As detailed in Table 1, we quantified the recovery of lymphocyte subsets by combining automated cell counting with multiparametric flow cytometry analysis. This calculation is based on the design of the leukocyte filters used in our study, each of which processes 400 mL of whole blood. Therefore, the filter is expected to capture leukocytes equivalent to those present in 400 mL of whole blood, and the recovery efficiency reflects the proportion successfully eluted in 40 mL of flushing solution. The recovery efficiency reflects the proportion of these captured cells successfully eluted in 40 mL of flushing solution, calculated as: (Cell number in 40 mL LF eluate/Cell number in 400 mL WB) × 100%. The cell number in 400 mL WB was derived by proportionally scaling the count from the 5 mL donor sample (i.e., Lymphocyte subset count in 5 mL WB × 80). Our results demonstrated significant differences in recovery efficiency among lymphocyte subpopulations (Table 1). The recovery efficiency of CD3^+^CD4^+^ T cells was significantly higher than that of NK cells (CD3^+^CD4^+^ T vs. NK, *P* < 0.001), and the recovery efficiency of NK cells was higher than that of CD3^+^CD8^+^ T cells (NK vs. CD3^+^CD8^+^ T, *P* = 0.0012).

**TABLE 1:** The recovery efficiency among lymphocyte subpopulations.

Group	40 mL LF (1 × 10^7^ cells)	400 mL WB (1 × 10^7^ cells)	Recovery efficiency (%)	Kruskal-Wallis test *P*-value	Dunn’s multiple comparisons test *P*-value
	Median (P_25_, P_75_)				
NK	1.42 (0.88, 2.09)	8.80 (5.64, 13.20)	16.30 (11.34, 24.7)	<0.0001	–
CD3^+^CD4^+^ T	11.28 (8.78, 14.12)	49.92 (39.16, 64.20)	22.02 (18.71, 27.21)		<0.0001
CD3^+^CD8^+^ T	1.92 (1.44, 2.63)	15.36 (11.22, 21.00)	13.00 (10.66, 16.75)		0.0012

Recovery efficiency = (Cell number in 40 mL LF eluate/Cell number in 400 mL WB) × 100%. This calculation is based on the design of the leukocyte filters used in our study, each of which processes 400 mL of whole blood. Therefore, the filter is expected to capture leukocytes equivalent to those present in 400 mL of whole blood, and the recovery efficiency reflects the proportion successfully eluted in 40 mL of flushing solution. LF, leukocyte filter; WB, whole blood.

### Influence of the age and gender of blood donors on recovered lymphocyte counts

**FIGURE 4:**
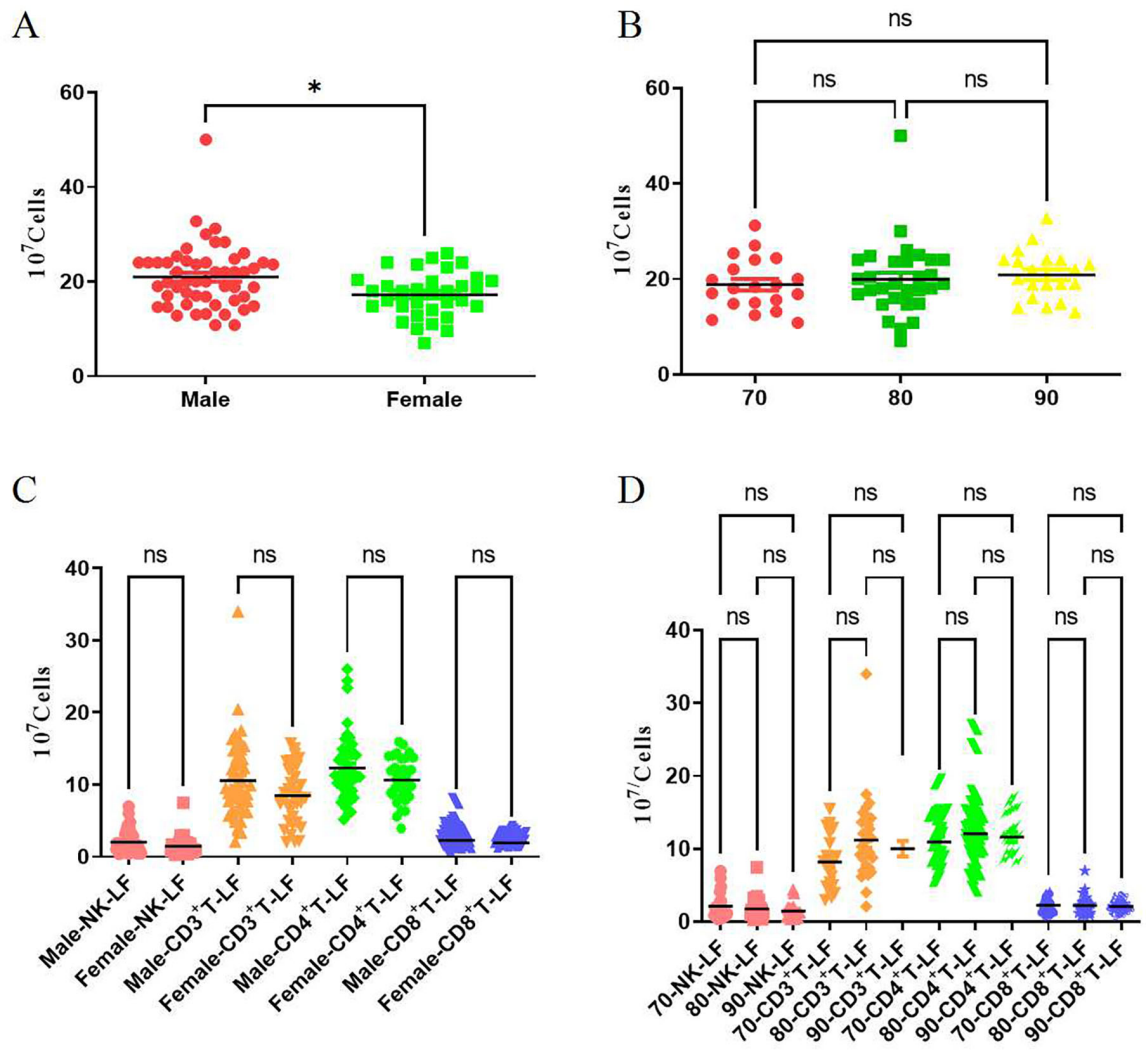
Effects of blood donors’ age and gender on the number of lymphocytes recovered from leukocyte filters. **(A)** Shows the comparison of total lymphocyte counts between different genders; **(B)** shows the comparison of total lymphocyte counts between different ages; **(C)** shows the comparison of counts of various lymphocyte subsets between different genders; **(D)** shows the comparison of counts of various lymphocyte subsets between different ages; LF stands for leukocyte filter; NK refers to CD3CD56^+^ NK; CD3^+^ T refers to CD3^+^ T cells; CD4^+^T refers to CD3^+^CD4^+^ T cell; CD8T refers to CD3^+^CD8^+^ T cell; the labels “70,” “80,” and “90” correspond to the birth cohorts of the 1970s, 1980s, and 1990s. ns indicates no significant difference (*P* > 0.05); *N* = 85, * indicates *P* < 0.05.

As shown in Figure 4, we found that the number of total lymphocytes in eluates was independent of the decade the donor was born in (70 vs. 80 vs. 90, *P* = 0.61 > 0.05, Figure 4B), but correlated with gender, with the number of total lymphocytes recovered from the leukocyte filters being significantly higher in males than in females (Female vs. Male, *P* < 0.05, Figure 4A).

By analyzing the number of recovered lymphocytes, we found that the number of NK cells and T cells (either CD3^+^CD4^+^ T cells or CD3^+^CD8^+^ T cells) in eluates was not related to the age and gender of blood donors (*P* > 0.05, Figures 4C, D).

### Transcriptome-based functional analysis

**FIGURE 5:**
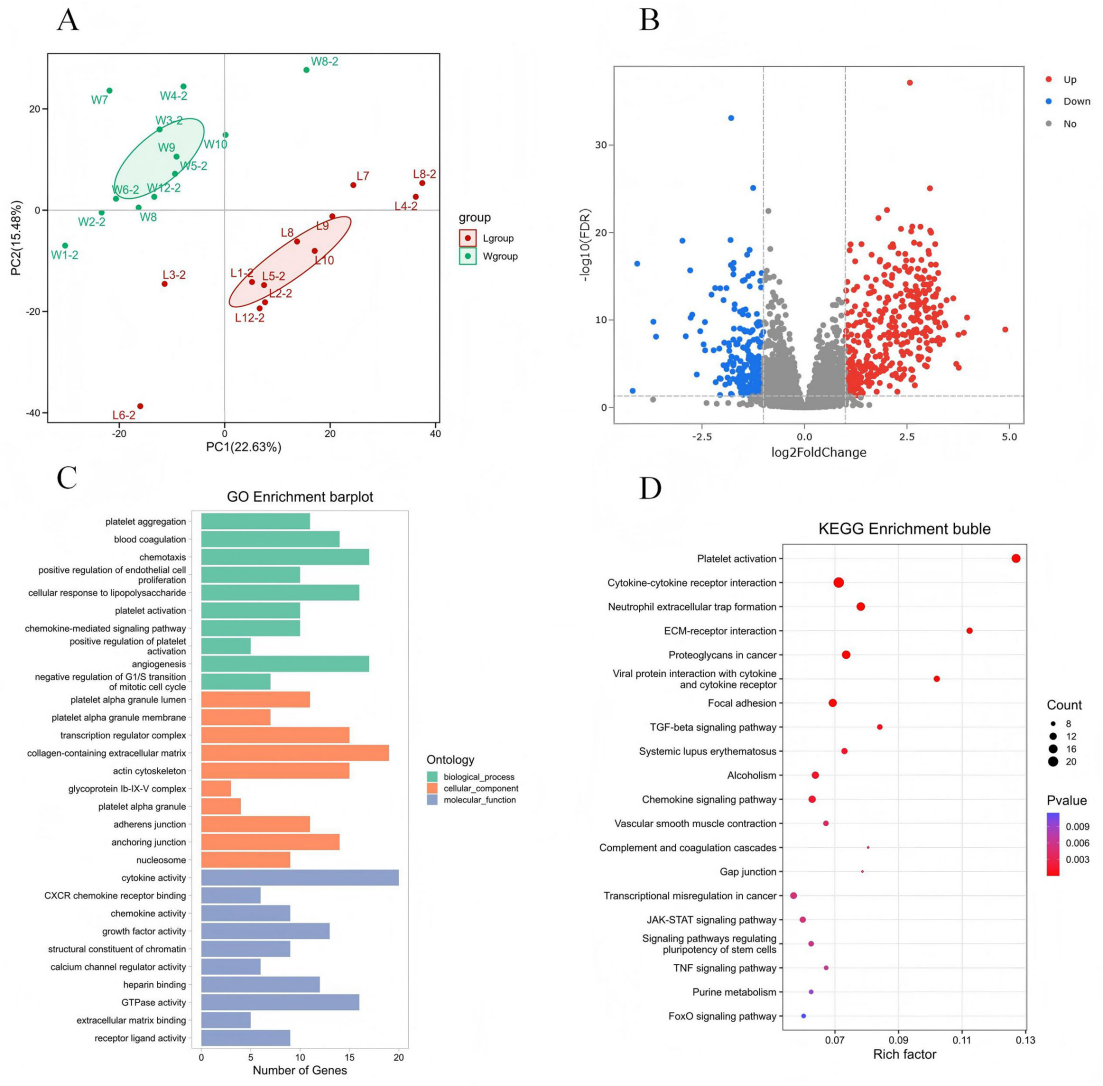
Differentially expressed genes and enrichment analysis of PBMCs derived from leukocyte filters and whole blood samples. **(A)** Shows the PCA of two groups; **(B)** shows the statistics of the number of differentially expressed genes; **(C,D)** shows the results of GO/KEGG enrichment analysis.

Supplementary Figure 2 presents the RNA electrophoresis results, confirming sample quality prior to library construction. Principal component analysis (PCA) showed clear separation between filter-derived and whole blood-derived PBMCs (Figure 5A). Transcriptome analysis identified 634 differentially expressed genes (402 upregulated, 232 downregulated) between filter-derived and whole blood PBMCs (Figure 5B, *N* = 12).

Gene Ontology enrichment analysis showed that the differentially expressed genes were enriched in biological processes such as angiogenesis, chemotaxis, and cellular response to lipopolysaccharide pathways. Among these, the up-regulated genes were mainly enriched in the chemotaxis pathway, while the down-regulated genes were mainly enriched in the positive regulation of cell migration pathway (Figure 5C).

Kyoto Encyclopedia of Genes and Genomes pathway analysis revealed enrichment in cytokine-cytokine receptor interaction, platelet activation, and neutrophil extracellular trap formation. Upregulated genes concentrated in the focal adhesion, while downregulated genes concentrated in cytokine-cytokine receptor interaction (Figure 5D).

Among 154 significantly enriched GO terms and 9 KEGG pathways (*P*-adjust < 0.05), none showed direct association with NK cell or T cell effector functions (*P*-adjust > 0.05 for all lymphocyte-specific terms). Furthermore, a targeted analysis for pathways related to apoptosis and programmed cell death revealed no significant enrichment (*P*-adjust > 0.05), indicating that the recovery process does not broadly induce transcriptional programs associated with cell death.

## Discussion

With the rapid advancement of cell therapy, the demand for immune cells in both clinical and basic research continues to grow. An increasing number of hospitals and research institutions require healthy immune cells as essential starting materials for therapeutic development. Studies have shown that, following *in vitro* modification, T cells from young, healthy allogeneic donors can exhibit superior anti-tumor efficacy compared to those derived from patients themselves (19). Early-phase trials have also confirmed the safety of allogeneic NK cell infusions (4), thereby supporting the feasibility of allogeneic cell therapy approaches.

Leukocyte filters used in blood banks represent a promising and sustainable source of such immune cells. The use of peripheral blood mononuclear cells (PBMCs) recovered from these filters eliminates the need to recruit additional healthy donors, thereby conserving resources. Moreover, these cells are inherently safe for research use since they have already undergone screening for transfusion-transmitted infections. Importantly, repurposing cells from used leukocyte filters also contributes to the reduction of biological waste (14). Nonetheless, their application in cell therapy remains underexplored, largely due to insufficient systematic data on recovery efficiency and functional compatibility.

Multiple studies have investigated methodologies for cell recovery from leukocyte filters (20). In our findings, where reverse flushing yielded higher lymphocyte recovery compared to a forward-then-reverse protocol which diverges from some previous reports (21). Beyond the flushing technique, the composition of the rinsing solution also plays a critical role. While some studies recommend the use of 1 × PBS supplemented with EDTA and dextran 40 for optimal recovery (22), our data align with other domestic studies (11) in showing no significant difference in lymphocyte yield between 1 × PBS and normal saline (NS) under reverse flushing conditions, supporting NS as a more cost-effective alternative. In terms of flushing volume, our evaluation of 20–80 mL eluates indicated that cell recovery improves with increasing volume up to 80 mL without a clear plateau. However, a volume of 40 mL was identified as optimal for balancing yield and cost-effectiveness, as it provided a yield not significantly different from 60 to 80 mL, consistent with prior recommendations (23).

Previous reports have described the proportions of T cells and NK cells recoverable from different filter types, with considerable variation observed across studies (22). In our study, the overall proportions of CD3^+^T cells and NK cells in leukocyte filter-derived lavage fluid did not differ significantly from those in whole blood (Figure 2), consistent with several earlier findings. However, within the CD3^+^T cell compartment, we observed a significant shift in the CD4^+^ to CD8^+^T subset ratio. This does not reflect a contradiction in data, but rather suggests a selective retention or release process during filtration. We hypothesize that distinct physical properties–such as cell size, surface adhesion molecule expression, or migratory capacity–may cause CD4^+^ and CD8^+^T cell subsets to interact differently with the filter matrix. For instance, certain CD8^+^T cell subsets may be more readily retained due to stronger adhesion, resulting in their lower relative recovery. This notion is supported by a study from Boudreau et al. (24), which reported filter-induced alterations in CD62L^+^ T-cell subsets. Although the specific subsets affected differ, their findings align with the general principle that the recovery process is not phenotypically neutral and can reproducibly reshape lymphocyte composition irrespective of the filter model used. This altered CD4/CD8 ratio indicates that filters may not be equivalent to whole blood for certain functional assays sensitive to subset composition, such as antigen-specific T cell responses measured by ELISpot.

NK cells and T cells are among the most widely utilized immune subsets in cell therapy research and development. A fundamental requirement for their application is that the initial cell number must be sufficient to support *in vitro* expansion. Previous studies suggest that 60–80 mL of whole blood provides an adequate number of cells for therapeutic use (17), and that approximately 45 mL of whole blood is sufficient to meet the input requirements of various NK and T cell culture systems (18). In our study, the absolute counts of lymphocyte subsets recovered from 40 mL of filter eluate showed no significant difference from those obtained from 80 mL of whole blood (Supplementary Figure 3, *P* > 0.05). These results confirm that the yield of immune cells recovered from leukocyte filters is sufficient to meet the demands of both basic research and clinical-scale applications, reinforcing the feasibility of this cell source.

While the adequacy of cell numbers has been established, the functional competence of filter-derived cells required further assessment. In this regard, Moazzeni et al. (13) successfully generated chimeric antigen receptor (CAR)-NK cells from filter-derived NK cells, which exhibited potent tumor-killing activity–directly supporting the therapeutic potential of this cell source. Similarly, Boudreau et al. (24) demonstrated that T cells recovered from leukoreduction systems can be effectively expanded and engineered into T-cell therapeutics, displaying robust proliferation and phenotypic stability. Our study adds to this body of evidence by being the first to compare the transcriptomic profiles of filter-derived and whole blood-derived PBMCs. We found no significant disruption in major functional pathways related to NK or T cell activity (FDR > 0.05). This transcriptomic equivalence, together with the proven expandability and engineerability of filter-derived cells (13, 24), strongly supports their suitability for adoptive cell therapies, including CAR-T and CAR-NK applications.

To comprehensively assess the functional integrity of filter-derived lymphocytes, we conducted transcriptome sequencing on PBMCs isolated from both leukocyte filter and whole blood samples. While 634 differentially expressed genes (DEGs) were identified, the subsequent GO and KEGG enrichment analyses yielded critical insight: none of the significantly enriched pathways were directly associated with the core effector functions of T cells or NK cells (*P*-adjust > 0.05). This finding is highly reassuring, as it indicates that the process of filtration and recovery does not systematically alter the transcription of genes governing critical lymphocyte processes such as cytotoxicity, cytokine production, or T cell receptor signaling.

Although our transcriptomic analysis identified enrichment in broad signaling pathways such as “Cytokine-cytokine receptor interaction,” “TNF signaling pathway,” and “JAK-STAT signaling pathway”–all of which can be associated with immune cell activation–this finding must be interpreted in the context of our experimental system. The enrichment was derived from bulk RNA-seq data of heterogeneous PBMC populations. It is therefore plausible that this signature reflects a generalized stress or activation response across multiple cell types, including monocytes and granulocytes, following their interaction with the leukocyte filter. Crucially, when our analysis focused specifically on gene sets defining core lymphocyte effector functions (e.g., T cell receptor signaling pathway, NK cell-mediated cytotoxicity), no significant enrichment was observed after multiple-testing correction. This dichotomy suggests that the recovery process induces a non-specific inflammatory milieu without selectively activating the key functional programs of T and NK cells. It should be noted that this study assessed the immediate transcriptomic impact of the filter recovery process, simulating a scenario in which cells are used directly without a resting phase. Future studies examining the effect of a resting period on transcriptomic normalization would be of great value.

This study has several limitations. All leukocyte filters used in this study were from a single manufacturer (Sichuan Nanger Biological Technology Co., LTD.). Given that filter structure and retention efficiency vary by brand (25), recovery outcomes may differ when using other products. Future work should include multiple filter types to enable cross-comparison of recovery performance and functional attributes. Furthermore, although our transcriptomic analysis revealed no major functional differences in PBMCs, the use of bulk RNA-seq represents a methodological constraint. This approach averages gene expression across all PBMCs and may mask subset-specific expression changes in purified NK or T cells. Subsequent studies employing single-cell RNA sequencing or transcriptomic analysis of sorted lymphocyte subsets could provide more precise insights into the functional equivalence of filter-derived immune cells.

In conclusion, NK cells and T cells recovered from leukocyte filters are suitable for both basic research and clinical applications. They are obtainable in sufficient quantities for *in vitro* expansion and demonstrate functional profiles comparable to those of peripheral blood-derived lymphocytes, supporting their use as a practical and sustainable alternative cell source for immunotherapy.

## Data Availability

The original contributions presented in this study are included in this article/Supplementary material, further inquiries can be directed to the corresponding authors.

## Supplementary material

Supplementary Figure 1 Representative gating strategy for the identification of lymphocyte subsets by flow cytometry. The gating strategy for lymphocyte immunophenotyping is shown. **(A)** Represents the proportion of lymphocytes. **(B)** Identifies CD3^–^CD56^+^ NK cells (Q1) and CD3^+^ T cells (Q3) from the lymphocyte population. **(C)** Then displays the subsequent analysis of the CD3^+^ T cells (from S2B, Q3), showing the CD3^+^CD4^+^ (Q3) and CD3^+^CD8^+^ (Q1) T-cell subsets. This sequential approach delineates the identification of specific lymphocyte populations.

Supplementary Figure 2 Assessment of RNA integrity by gel electrophoresis.

Supplementary Figure 3 Quantitative comparison of lymphocyte subsets recovered from 40 mL leukocyte filter (LF) eluates versus 80 mL whole blood sample (WB). ns indicates no significant difference (*P* > 0.05); NK refers to CD3^–^CD56^+^ NK; T refers to CD3^+^ T cells; CD4^+^ T-LF refers to CD3^+^CD4^+^ T cell derived from leukocyte filters; CD4^+^ T-WB refers to CD3^+^CD4^+^ T cell derived from whole blood samples; CD8^+^T-LF refers to CD3^+^CD8^+^ T cell derived from leukocyte filters; CD8^+^T-WB refers to CD3^+^CD8^+^ T cell derived from whole blood samples. ns indicates no significant difference (*P* > 0.05).
