# Supplementary figures and images for: Recovery efficiency and functional characterization of T cells and NK cells from leukocyte filters
Source: Front Med (Lausanne). 2025 Nov 28;12:1681146. doi: 10.3389/fmed.2025.1681146 (PMC12698533; doi:10.3389/fmed.2025.1681146)

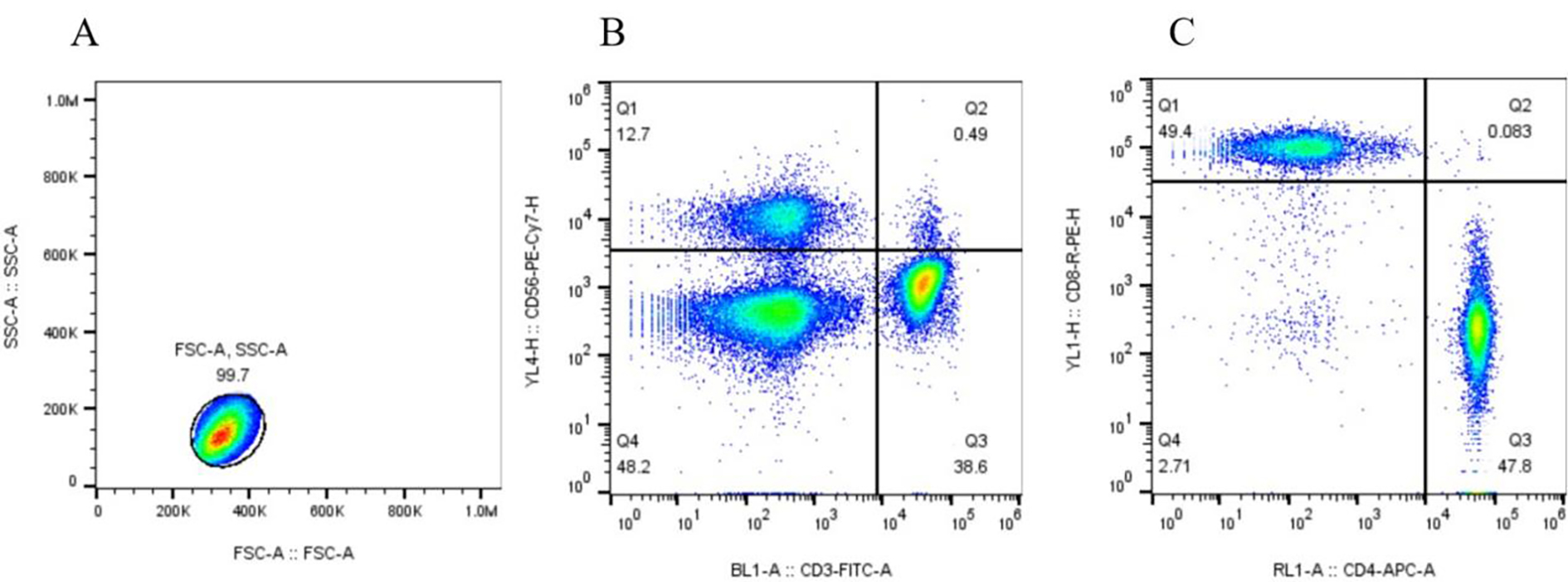

Supplement: Supplementary Figure 1 — Representative gating strategy for the identification of lymphocyte subsets by flow cytometry. The gating strategy for lymphocyte immunophenotyping is shown. (A) Represents the proportion of lymphocytes. (B) Identifies CD3–CD56+ NK cells (Q1) and CD3+ T cells (Q3) from the lymphocyte population. (C) Then displays the subsequent analysis of the CD3+ T cells (from S2B, Q3), showing the CD3+CD4+ (Q3) and CD3+CD8+ (Q1) T-cell subsets. This sequential approach delineates the identification of specific lymphocyte populations. [file Image_1.jpeg]

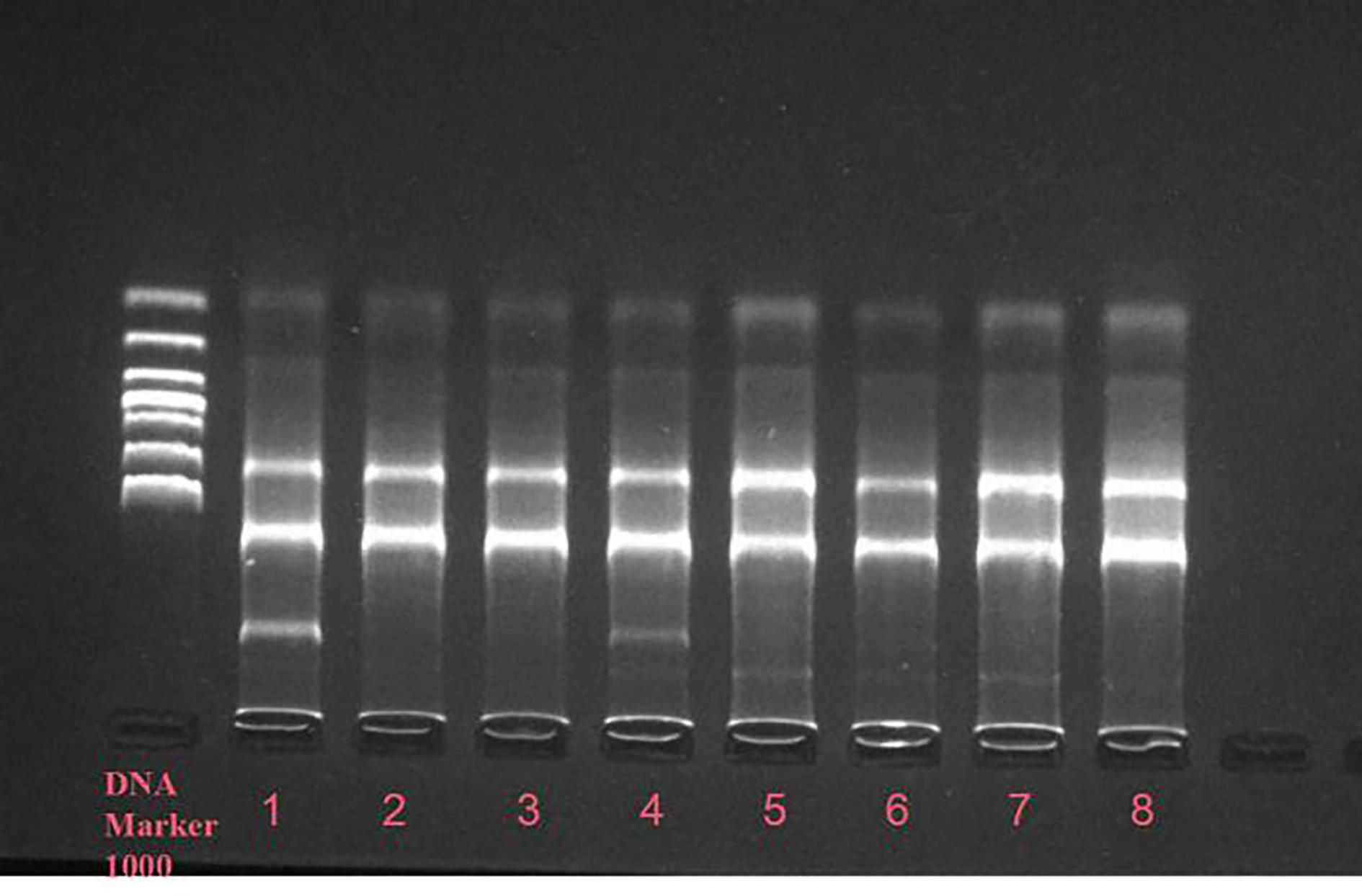

Supplement: Supplementary Figure 2 — Assessment of RNA integrity by gel electrophoresis. [file Image_2.jpeg]

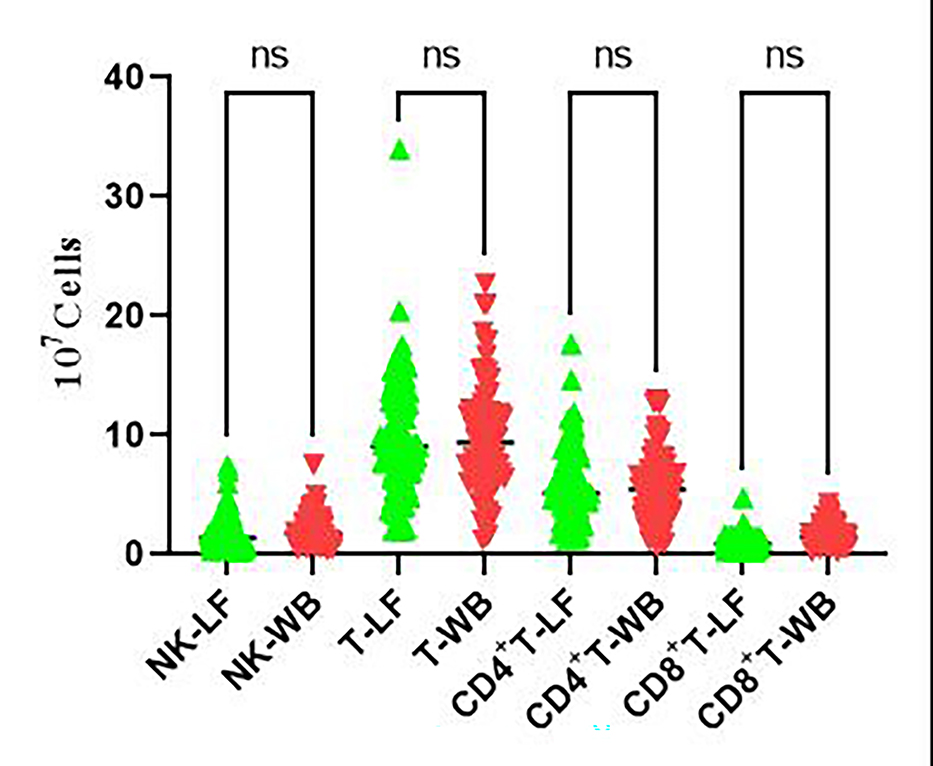

Supplement: Supplementary Figure 3 — Quantitative comparison of lymphocyte subsets recovered from 40 mL leukocyte filter (LF) eluates versus 80 mL whole blood sample (WB). ns indicates no significant difference (P > 0.05); NK refers to CD3–CD56+ NK; T refers to CD3+ T cells; CD4+ T-LF refers to CD3+CD4+ T cell derived from leukocyte filters; CD4+ T-WB refers to CD3+CD4+ T cell derived from whole blood samples; CD8+T-LF refers to CD3+CD8+ T cell derived from leukocyte filters; CD8+T-WB refers to CD3+CD8+ T cell derived from whole blood samples. ns indicates no significant difference (P > 0.05). [file Image_3.jpeg]
